# Estimated glucose disposal rate mediates the association between Life’s Crucial 9 and congestive heart failure: a population-based study

**DOI:** 10.3389/fendo.2025.1540794

**Published:** 2025-04-03

**Authors:** Liping Wang, Yaying Xu, Lele Chen, Huifeng Zhang

**Affiliations:** ^1^ Department of Cardiovascular, The First Affiliated Hospital, and College of Clinical Medicine of Henan University of Science and Technology, Luoyang, China; ^2^ Department of Endocrinology, The First Affiliated Hospital, and College of Clinical Medicine of Henan University of Science and Technology, Luoyang, China; ^3^ Henan Provincial People's Hospital, Zhumadian, China

**Keywords:** Life’s Crucial 9 (LC9), estimated glucose disposal rate (eGDR), Congestive Heart Failure (CHF), NHANES, cross-sectional study, mediation analysis

## Abstract

**Background:**

Life’s Crucial 9 (LC9) is the latest indicator of cardiovascular health (CVH), and the estimated glucose disposal rate (eGDR) is a non-invasive indicator of insulin resistance (IR). However, the relationships between LC9 and eGDR and congestive heart failure (CHF) remain unknown.

**Methods:**

In this cross-sectional study, participants aged ≥20 years in the NHANES database from 2005 to 2018 were analyzed. Weighted linear regression, logistic regression, subgroup analysis, and restricted cubic spline (RCS) analysis were employed to analyze the associations among LC9, eGDR, and CHF. Mediation analysis was used to explore the mediating role of eGDR in the association between LC9 and CHF.

**Results:**

A total of 22,699 adult participants were included, among whom 661 suffered from CHF. The mean age of the participants was 47.52 (0.26) years old, with 11186 (48.68%) males and 11513 (51.32%) females. The average value of LC9 was 71.16 (0.22), and that of eGDR was 7.91 (0.04). After adjusting for confounding factors, linear regression showed that LC9 was independently and positively associated with eGDR (β: 1.11, 95%CI: 1.07 - 1.14, P < 0.0001). Logistic regression indicated that both LC9 (OR: 0.76, 95%CI: 0.65 - 0.88, P < 0.001) and eGDR (OR: 0.81, 95%CI: 0.76 - 0.86, P < 0.0001) were independently and negatively associated with the prevalence of CHF. Mediation analysis revealed that the association between LC9 and CHF was mainly mediated by eGDR, with a proportion of 66%.

**Conclusion:**

This study suggests that higher LC9 scores and eGDR values imply a lower prevalence of CHF. Meanwhile, eGDR is the main intermediate factor in the association between LC9 and CHF, indicating that good CVH may reduce the prevalence of CHF by improving IR.

## Introduction

Cardiovascular disease (CVD) plays a major role among the fatal diseases globally, and congestive heart failure (CHF) is the terminal manifestation of the progression of CVD (1). Worldwide, at least 1 to 2 out of every 100 people suffer from CHF, and the prevalence of CHF is even higher among the elderly population, with approximately 1 out of every 10 people having CHF (2, 3). In the United States, around 6 million adults are affected by heart failure (HF) (4). Currently, CHF may not be curable. The existing treatment methods can only improve the quality of life and prolong the survival period; nevertheless, the prognosis remains extremely poor (5). Therefore, preventing the occurrence of CHF has become an important task for medical workers.

In 2022, the American Heart Association (AHA) released the cardiovascular health (CVH) indicator “Life’s Essential 8” (LE8), which combines eight aspects including diet, physical activity, nicotine exposure, sleep health, body mass index, blood lipids, blood glucose, and blood pressure (6). Studies have shown that among American adults, CHF is the second most relevant non-communicable disease (NCD) associated with LE8 (7). This highlights the importance of evaluating LE8 scores in the prevention and early intervention of CHF. In 2024, the AHA updated the CVH indicator and introduced Life’s Crucial 9 (LC9) after incorporating mental health into the assessment items (8). Ge et al. (9) were the first to evaluate that LC9, which includes mental health, has an advantage over LE8 in predicting the risks of all-cause and cardiovascular-specific mortality in the general population. Gong et al. (10) found that there is a negative association between LC9 and overactive bladder syndrome. However, the relationship between LC9 and CHF has not yet been revealed.

There is a complex interaction between diabetes mellitus (DM) and HF. The risk of HF in patients with DM is significantly increased, and the longer the duration of DM, the higher the incidence of HF (11). A number of studies have shown that DM is an important independent risk factor for heart failure. The incidence of HF in DM patients is significantly higher than that in non-DM patients (12–14). In the Framingham Heart Study, DM increased the risk of new-onset HF by twofold in men and fourfold in women respectively (15). Previous studies have also shown that the triglyceride-glucose (TyG), an alternative indicator of insulin resistance (IR), is an independent risk factor for new-onset HF in the general population (16). The estimated glucose disposal rate (eGDR), which combines waist circumference (WC), blood pressure, and glycated hemoglobin (HbA1c), has been proven to be a more valuable alternative indicator of IR in type 1 diabetes mellitus (T1DM). However, the relationship between eGDR and CHF has not been reported yet.

There is evidence showing that Life’s Simple 7 (LS7), the cardiovascular health (CVH) indicator first released by the AHA, can improve IR (17). A higher eGDR represents a lower level of IR in the body. Therefore, we speculate that LC9 may be positively associated with eGDR. In addition, given that IR is a risk factor for HF, we also speculate that eGDR may act as a mediating factor between LC9 and CHF. Up to now, there has been scarcely any research exploring how LC9, which incorporates multiple lifestyle and health factors, interacts with eGDR, an alternative indicator of IR, and ultimately affects the high-risk status of CVH. Our study, by leveraging the large cross-sectional data from National Health and Nutrition Examination Survey (NHANES) and with an eye on the potential extrapolation to China’s national conditions in the future, aims to fill this gap. By delving into these relationships, we expect to not only provide novel insights for the diagnosis and prevention of CHF but also lay the foundation for future targeted interventions, hopefully reducing the burden of cardiovascular diseases in China and even globally.

## Methods

### Study participants

The data sources of the NHANES are extensive and diverse, integrating information from multiple fields such as participants’ demographic details, diet and nutrition, physical measurements, physiological and biochemical indicators, disease history, and lifestyle. These data are collected through multiple means, including questionnaires, physical examinations, and laboratory tests. Moreover, it has long-term tracking data since 1960. Its sample collection employs a stratified multistage probability sampling method, with the sample updated every 2 - 4 years. The age range covers all age groups from infants to the elderly, encompassing all major ethnic and racial groups in the United States. During the collection process, investigators first conduct face-to-face questionnaires to gather information about individuals, their families, lifestyle habits, diet, etc. Then, professional medical staff perform comprehensive physical examinations to measure multiple physical indicators. Finally, biological samples are collected and sent to professional laboratories for biochemical indicator testing, ensuring an accurate reflection of the health and nutrition status of the US population and its dynamic changes. For more details, please refer to https://wwwn.cdc.gov/nchs/nhanes/Default.aspx.

**Figure 1:**
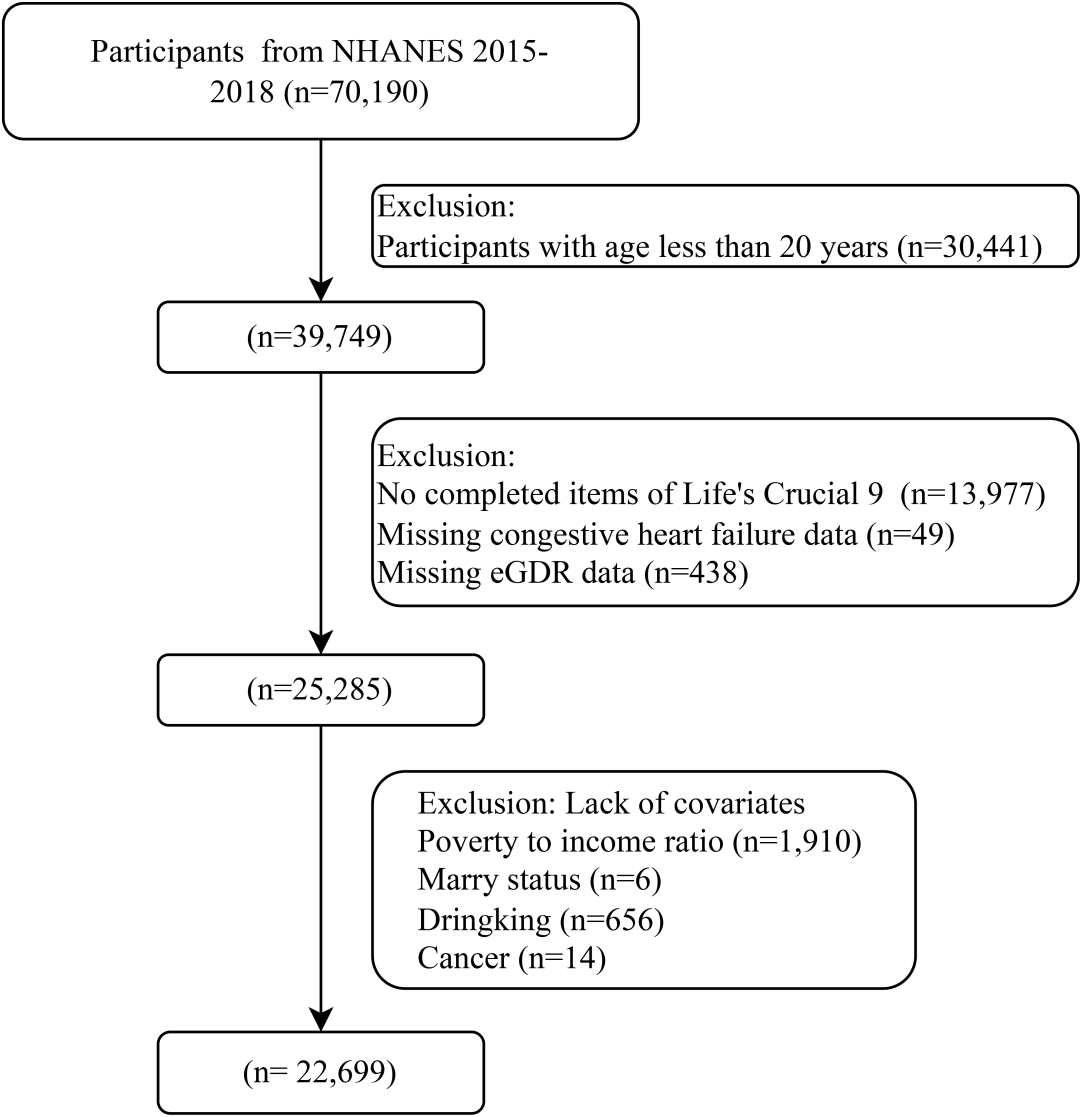
Flow chart of participant recruitment.

The data of this cross-sectional study were sourced from participants of the NHANES across a total of seven cycles from 2005 to 2018. This study has obtained ethical approval from the Ethics Review Committee of the National Center for Health Statistics. Before participating in the study, all individuals received detailed written information about the study and signed written informed consent forms. Across the seven cycles, there were initially 70,190 participants. Subsequently, the sample was screened based on a series of exclusion criteria. Firstly, since our study focused on the adult population, and the physiological and health characteristics of minors under 20 years old might differ significantly from those of adults, potentially affecting the study results, 30,441 participants under 20 years old were excluded. Secondly, 13,977 participants without complete data for all nine items of LC9 were removed. The integrity of LC9 data is crucial as it comprehensively reflects multiple aspects of cardiovascular health, and incomplete data could lead to inaccurate analysis. Next, given that CHF and eGDR are central to our research questions, 49 participants with missing CHF data and 438 participants with missing eGDR values were excluded. Finally, 2,586 participants with missing covariate data were also excluded, as covariates play an essential role in controlling for confounding factors and ensuring the validity of our statistical analyses. After these exclusions, a final sample of 22,699 participants was retained for further analysis (**Figure 1**).

### LC9

LC9 was constructed on the basis of LE8. LE8 takes into account eight factors, including four healthy behaviors (diet, physical activity, nicotine exposure, and sleep duration) and four health factors (body mass index, non-high-density lipoprotein cholesterol, blood glucose, and blood pressure). The detailed scoring criteria for each factor are shown in **Supplementary Tables 1** and **2** (6). According to the study by Ge et al. (9), LC9 additionally incorporates a depression score. In this study, the depression score was calculated based on the Patient Health Questionnaire-9 (PHQ-9) score. A higher PHQ-9 score indicates a higher level of current depressive symptoms. The depression scores were divided into 100, 75, 50, 25, and 0, corresponding to the PHQ-9 scores of 0 to 4, 5 to 9, 10 to 14, 15 to 19, and 20 to 27 respectively (18). The LC9 score was calculated as the arithmetic mean of the nine factors (9).

### Diagnosis of CHF

The diagnosis of CHF was determined according to the Medical Conditions Questionnaire (MCQ), and this diagnostic method has been supported by many literatures (19, 20). The researchers asked the participants the following question: “Has a doctor ever told you that you have congestive heart failure?” Participants who answered “yes” were classified as having CHF.

### eGDR

As an alternative indicator of IR, the eGDR was calculated using the following formula: eGDR=21.158−0.09*WC−3.407*Hypertension−0.551*HbA1c(%) . In the formula, WC represents waist circumference, with the unit being centimeters; the value of hypertension was defined as 1 if suffering from hypertension, and 0 otherwise (21). WC was collected by trained health technicians at the Mobile Examination Center (MEC). During the physical measurement examination, the health technicians were assisted by recorders. Blood specimens were processed, stored and shipped to Fairview Medical Center Laboratory at the University of Minnesota, Minneapolis Minnesota for analysis. Detailed specimen collection and processing instructions are discussed in the NHANES LPM. In this assay, the stable (SA1c) and labile (LA1c) A1c forms can be individually resolved on the chromatogram without manual pretreatment, allowing accurate measurement of the stable form of HbA1c. The analyzer dilutes the whole blood specimen with a hemolysis solution, and then injects a small volume of the treated specimen onto the HPLC analytical column. Separation is achieved by utilizing differences in ionic interactions between the cation exchange group on the column resin surface and the hemoglobin components. The hemoglobin fractions (A1c, A1b, F, LA1c, SA1c, A0 and H-Var) are subsequently removed from the column material by step-wise elution using elution buffers each with a different salt concentration. The separated hemoglobin components pass through the photometer flow cell where the analyzer measures changes in absorbance at 415 nm. The analyzer integrates and reduces the raw data, and then calculates the relative percentages of each hemoglobin fraction. There were changes to the equipment from NHANES 2005-2006 to NHANES 2007-2008. For NHANES 2005-2006, glycohemoglobin measurements were performed on the A1c 2.2 Plus Glycohemoglobin Analyzer (Tosoh Medics, Inc., 347 Oyster Pt. Blvd., Suite 201, So. San Francisco, Ca 94080.). For NHANES 2007-2008 glycohemoglobin measurements were performed on the A1c G7 HPLC Glycohemoglobin Analyzer (Tosoh Medics, Inc., 347 Oyster Pt. Blvd., Suite 201, So. San Francisco, Ca 94080.). (https://wwwn.cdc.gov/Nchs/Data/Nhanes/Public/2011/DataFiles/GHB_G.htm#LBXGH). Hypertension was defined as the self-report hypertension, or systolic blood pressure ≥140 mmHg, or diastolic blood pressure ≥90 mmHg, or taking antihypertensive drugs (22).

### Covariates

All covariates included demographic, socioeconomic, lifestyle, and health-related characteristics (**Supplementary Table 3**). Demographic variables and socioeconomic variables were collected: sex (male/female), age (continuous), race (non-Hispanic white, non-Hispanic black, Mexican American, other Hispanic, and multiracial), educational background (college degree or above and others), marital status (married, unmarried, divorced), and poverty income ratio (PIR) (<1.3, 1.3 - 3.5, >3.5). Lifestyle and health-related variables were collected: smoking (never/former/current), alcohol consumption status (never/former/current), physical activity (<700 minutes/week, 700 - 2400 minutes/week, >2400 minutes/week, not available), dietary energy intake (continuous), body mass index (BMI) (non-overweight, overweight or obese). In addition, health-related characteristics were considered: cancer (yes/no), hyperlipidemia (yes/no), atherosclerotic cardiovascular disease (ASCVD) (yes/no), and DM (yes/no).

### Statistical analysis

Adhering to the principle of the complex sampling design adopted by NHANES, this study used the MEC weights (1/7 * WTMEC2YR) for subsequent analyses. Firstly, we divided the participants into two groups according to whether they had CHF or not. Continuous variables were expressed as means and standard errors (SEs), while categorical variables were presented in terms of the number of cases (N) and percentages (%). To determine the differences between the two groups, a t-test was conducted for continuous variables, and a chi-square test was used for categorical variables.

Firstly, the effect (β) and 95% confidence intervals (CIs) of LC9 on eGDR were statistically analyzed through weighted linear regression. Multiple models were constructed. The crude model (Model 0) did not adjust for any variables. Model 1 adjusted for age, sex, race, marital status, PIR, and educational level; Model 2 further adjusted for alcohol consumption status and dietary energy on the basis of Model 1. Model 3 further adjusted for cancer, hyperlipidemia, and ASCVD on the basis of Model 2.

Secondly, the odds ratios (ORs) and 95% CIs of the association between eGDR and CHF were statistically analyzed through weighted logistic regression. Four models were also constructed. The crude model (Model 0) did not adjust for any variables. Model 1 adjusted for age, sex, race, marital status, PIR, and educational level; Model 2 further adjusted for smoking, alcohol consumption, physical activity, dietary energy, and BMI on the basis of Model 1. Model 3 further adjusted for cancer, DM, hyperlipidemia, and ASCVD on the basis of Model 2.

In addition, the ORs and 95% CIs of the association between LC9 and CHF were statistically analyzed through weighted logistic regression. Four models were also constructed in the same way. The crude model (Model 0) did not adjust for any variables. Model 1 adjusted for age, sex, race, marital status, PIR, and educational level; Model 2 further adjusted for alcohol consumption and dietary energy on the basis of Model 1. Model 3 further adjusted for cancer, hyperlipidemia, and ASCVD on the basis of Model 2. The collinearity of the above numerous models was determined by the variance inflation factor (VIF). In this study, all VIFs were less than 10.

To better evaluate the relationships among exposure, outcome, and mediator factors, this study also used weighted restricted cubic spline (RCS) to fit the dose-response relationships between LC9 and eGDR, LC9 and CHF, as well as eGDR and CHF.

Finally, mediation analysis was conducted. The bootstrap method with 5,000 exchanges was adopted to estimate the mediation proportion of eGDR in the association between LC9 and CHF.

All statistical implementations were achieved using R version 4.44. The main packages used included the “survey” package, the “rms” package, the “mediation” package, and the “tableone” package.

## Results

### Population characteristics

A total of 22,699 participants aged over 20 years were included (**Figure 1**). Among these 22,699 participants, 661 were diagnosed with CHF. The mean age of the participants was 47.52 (0.26) years old, with 11186 (48.68%) males and 11513 (51.32%) females. The average value of LC9 was 71.16 (0.22), and that of eGDR was 7.91 (0.04). **Table 1** presents the characteristics of the participants in the CHF group and the non-CHF group. Compared with the non-CHF group, the CHF group had lower LC9 scores and lower eGDR values. In addition, the CHF group had a higher proportion of older individuals, males, non-Hispanic blacks, those with a low educational level, divorced people, and the poor. Moreover, the CHF group was more likely to have a history of alcohol consumption and smoking, to be overweight or obese, and to suffer from ASCVD, DM, cancer, and hyperlipidemia.

**Table 1:** Weighted baseline characterization.

Characteristics	Total (N=22,699)	Without CHF (N=22,038)	With CHF (N=661)	*P*-value
LC9 score, Mean (S.E.)	71.16 (0.22)	71.40 (0.22)	59.73 (0.76)	< 0.0001
eGDR, Mean (S.E.)	7.91 (0.04)	7.97 (0.04)	5.10 (0.13)	< 0.0001
Age, Mean (S.E.)	47.52 (0.26)	47.12 (0.26)	66.21 (0.59)	< 0.0001
Sex, n (%)				0.004
Female	11513 (51.32)	11250 (51.47)	263 (44.11)	
Male	11186 (48.68)	10788 (48.53)	398 (55.89)	
Race, n (%)				< 0.0001
Mexican American	3333 (7.47)	3283 (7.56)	50 (3.49)	
Non-Hispanic Black	4552 (9.73)	4393 (9.66)	159 (13.12)	
Non-Hispanic White	10637 (71.54)	10259 (71.44)	378 (76.53)	
Other Hispanic	2018 (4.84)	1971 (4.87)	47 (3.44)	
Other Race - Including Multi-Racial	2159 (6.42)	2132 (6.48)	27 (3.42)	
Educational level, n (%)				< 0.0001
No college	10052 (36.40)	9663 (35.99)	389 (55.37)	
College or equivalent	12647 (63.60)	12375 (64.01)	272 (44.63)	
Marital status, n (%)				< 0.0001
Divorced or separated or widowed	4844 (17.68)	4598 (17.35)	246 (33.53)	
Never married	3981 (16.94)	3938 (17.17)	43 (6.09)	
Already married or cohabitation	13874 (65.38)	13502 (65.48)	372 (60.37)	
Poverty to income ratio, n (%)				< 0.0001
<1.3	6576 (18.81)	6325 (18.62)	251 (27.79)	
1.3–3.5	8613 (35.52)	8322 (35.20)	291 (50.11)	
>3.5	7510 (45.68)	7391 (46.18)	119 (22.10)	
Drinking status, n (%)				< 0.0001
Former drinker	3684 (13.28)	3462 (12.85)	222 (33.55)	
Never drinked	2904 (9.94)	2818 (9.89)	86 (12.31)	
Current drinker	16111 (76.78)	15758 (77.26)	353 (54.14)	
Smoking status, n (%)				< 0.0001
Never smoked	12470 (55.21)	12224 (55.62)	246 (36.17)	
Former smoker	5681 (25.60)	5398 (25.20)	283 (44.29)	
Current smoker	4548 (19.19)	4416 (19.18)	132 (19.54)	
Physical activity (MET), minutes/week, n (%)				< 0.0001
<700	4537 (20.44)	4405 (20.45)	132 (20.20)	
700-2400	5211 (24.98)	5093 (25.10)	118 (19.45)	
>=2400	7581 (35.18)	7458 (35.53)	123 (18.49)	
Not report	5370 (19.40)	5082 (18.92)	288 (41.86)	
Dietary energy, Mean (S.E.)	2197.14 (9.54)	2203.81 (9.69)	1884.05 (46.50)	< 0.0001
Body mass index, n (%)				< 0.0001
<25 kg/m^2^	6514 (29.83)	6395 (30.10)	119 (17.54)	
>=25 kg/m^2^	16185 (70.17)	15643 (69.90)	542 (82.46)	
Hyperlipidemia, n (%)				< 0.0001
No	6444 (29.27)	6376 (29.69)	68 (9.56)	
Yes	16255 (70.73)	15662 (70.31)	593 (90.44)	
ASCVD, n (%)				< 0.0001
No	20565 (92.63)	20370 (93.97)	195 (29.77)	
Yes	2134 (7.37)	1668 (6.03)	466 (70.23)	
DM, n (%)				< 0.0001
DM	3614 (12.04)	3312 (11.37)	302 (43.31)	
IFG	1071 (4.74)	1043 (4.72)	28 (6.02)	
IGT	956 (3.77)	926 (3.74)	30 (5.31)	
No	17058 (79.45)	16757 (80.17)	301 (45.35)	
Cancer, n (%)				< 0.0001
No	20503 (89.89)	19986 (90.23)	517 (73.72)	
Yes	2196 (10.11)	2052 (9.77)	144 (26.28)	

SE, standard error; PIR, poverty-to-income ratio; MET, metabolic equivalent; LC9, Life’s Crucial 9; eGDR, estimated glucose disposal rate; ASCVD, atherosclerotic cardiovascular disease; CHF, congestive heart failure; DM, diabetes mellitus; IFG, impaired fasting glycaemia; IGT, impaired glucose tolerance.

### The association between LC9 and eGDR

**Table 2** presents the results of the weighted linear regression between LC9 and eGDR. When LC9 was included in the model as a continuous variable, in Model 0, an increase of 10 points in LC9 was associated with an increase of 1.21 in eGDR (β = 1.21, 95% CI: 1.18 – 1.23, P < 0.0001). In the fully adjusted Model 3, an increase of 10 points in LC9 was associated with an increase of 1.11 in eGDR (β = 1.11, 95% CI: 1.07 – 1.14, P < 0.0001). When LC9 was included in the model as a four-category variable, after full adjustment (Model 3), compared with the quartile 1 group, the eGDR in the quartile 2 group (β = 1.36, 95% CI: 1.26 – 1.46, P < 0.0001), the quartile 3 group (β = 2.23, 95% CI: 2.14 – 2.32, P < 0.0001), and the quartile 4 group (β = 3.19, 95% CI: 3.07 – 3.31, P < 0.0001) all increased significantly, and there was a trend of increasing eGDR among the four groups (P for trend < 0.0001).

**Table 2:** β estimates for the association between LC9 and eGDR.

		Model 0		Model 1		Model 2		Model 3	
		β (95%CI)	*P-*value	β (95%CI)	*P-*value	β (95%CI)	*P-*value	β (95%CI)	*P-*value
eGDR~LC9	Per 10	1.21 (1.18,1.23)	<0.0001	1.09 (1.06, 1.11)	<0.0001	1.24 (1.21, 1.27)	<0.0001	1.11 (1.07, 1.14)	<0.0001
	Quartile 1	reference		reference		reference		reference	
	Quartile 2	1.51 (1.39,1.63)	<0.0001	1.43 (1.32, 1.53)	<0.0001	1.62 (1.52, 1.72)	<0.0001	1.36 (1.26, 1.46)	<0.0001
	Quartile 3	2.59 (2.49,2.69)	<0.0001	2.36 (2.26, 2.45)	<0.0001	2.63 (2.54, 2.72)	<0.0001	2.23 (2.14, 2.32)	<0.0001
	Quartile 4	4.14 (4.04,4.25)	<0.0001	3.65 (3.55, 3.75)	<0.0001	3.68 (3.56, 3.79)	<0.0001	3.19 (3.07, 3.31)	<0.0001
	*P* for trend		<0.0001		<0.0001		<0.0001		<0.0001

Model 0: Crude model. Model 1: Adjusted for age, sex, race, marital status, education, and poverty-income ratio. Model 2: Additionally adjusted for drinking and total energy intake. Model 3: Further adjusted for ASCVD, cancer, and hyperlipidemia.

CI, confidence interval; LC9, Life’s Crucial 9; eGDR, estimated glucose disposal rate; ASCVD, atherosclerotic cardiovascular disease.

**Figure 2:**
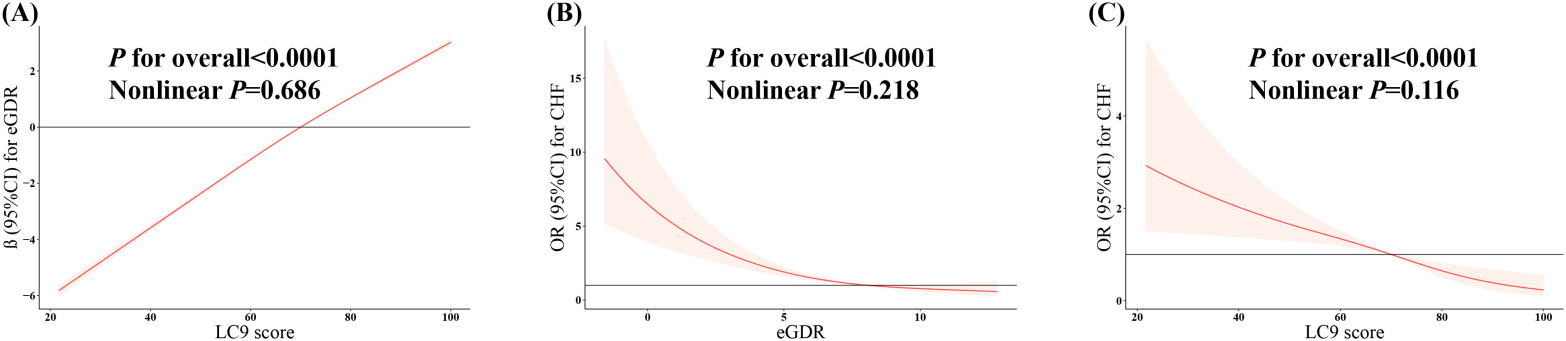
Weighted restricted cubic spline regression. **(A)** The dose-response relationship between LC9 and eGDR. Model was adjusted for age, sex, race, marital status, education, poverty-to-income ratio, drinking, total energy intake, ASCVD, cancer, and hyperlipidemia. **(B)** The dose-response relationship between eGDR and CHF. Model was adjusted for age, sex, race, marital status, education, poverty-to-income ratio, smoking, drinking, physical activity, dietary energy, BMI, cancer, DM, hyperlipidemia, and ASCVD. **(C)** The dose-response relationship between LC9 and CHF. Model was adjusted for age, sex, race, marital status, education, poverty-to-income ratio, drinking, total energy intake, ASCVD, cancer, and hyperlipidemia. LC9, Life’s Crucial 9; eGDR, estimated glucose disposal rate; ASCVD, atherosclerotic cardiovascular disease; CHF, congestive heart failure; BMI, body mass index; DM, diabetes mellitus.

As shown in **Figure 2A**, after fully adjusting for covariates, the RCS showed a strong linear positive association between LC9 and eGDR (P for nonlinear = 0.686).

### The association between eGDR and CHF

**Table 3** presents the results of the weighted logistic regression between eGDR and CHF. When eGDR was included in the model as a continuous variable, in Model 0, an increase of 1 in eGDR was associated with a 30% reduction in the odds of CHF (OR = 0.70, 95% CI: 0.67 – 0.72, P < 0.0001). In the fully adjusted Model 3, an increase of 1 in eGDR was associated with a 19% reduction in the odds of CHF (OR = 0.81, 95% CI: 0.76 – 0.86, P < 0.0001). When eGDR was included in the model as a four-category variable, after full adjustment (Model 3), compared with the quartile 1 group, the prevalence of CHF in the quartile 2 group (OR = 0.60, 95% CI: 0.44 – 0.81, P < 0.001), the quartile 3 group (OR = 0.40, 95% CI: 0.26 – 0.62, P < 0.0001), and the quartile 4 group (OR = 0.35, 95% CI: 0.19 – 0.63, P < 0.001) all decreased significantly, and there was a trend of decreasing prevalence of CHF among the four groups (P for trend < 0.0001).

**Table 3:** OR estimates for the association between eGDR and CHF.

		Model 0		Model 1		Model 2		Model 3	
		OR (95%CI)	*P-*value	OR (95%CI)	*P-*value	OR (95%CI)	*P-*value	OR (95%CI)	*P-*value
CHF~eGDR	Per 1	0.70 (0.67,0.72)	<0.0001	0.75 (0.72,0.79)	<0.0001	0.75 (0.71,0.79)	<0.0001	0.81 (0.76, 0.86)	<0.0001
	Quartile 1	reference		reference		reference		reference	
	Quartile 2	0.43 (0.34,0.55)	<0.0001	0.50 (0.38,0.65)	<0.0001	0.51 (0.39,0.67)	<0.0001	0.60 (0.44, 0.81)	<0.001
	Quartile 3	0.11 (0.07,0.16)	<0.0001	0.22 (0.15,0.34)	<0.0001	0.23 (0.15,0.35)	<0.0001	0.40 (0.26, 0.62)	<0.0001
	Quartile 4	0.05 (0.03,0.08)	<0.0001	0.19 (0.12,0.31)	<0.0001	0.19 (0.11,0.34)	<0.0001	0.35 (0.19, 0.63)	<0.001
	*P* for trend		<0.0001		<0.0001		<0.0001		<0.0001

Model 0: Crude model. Model 1: Adjusted for age, sex, race, marital status, education, and poverty-income ratio. Model 2: Additionally adjusted for smoking, drinking, physical activity, dietary energy, and BMI. Model 3: Further adjusted for ASCVD, cancer, and hyperlipidemia and DM.

OR, odds ratio; CI, confidence interval; eGDR, estimated glucose disposal rate; ASCVD, atherosclerotic cardiovascular disease; BMI, body mass index; CHF, congestive heart failure; DM, diabetes mellitus.

As shown in **Figure 2B**, after fully adjusting for covariates, the RCS showed a negative linear relationship between eGDR and CHF (P for nonlinear = 0.218).

### The association between LC9 and CHF

**Table 4** presents the results of the weighted logistic regression between LC9 and CHF. When LC9 was included in the model as a continuous variable, in Model 0, an increase of 10 points in LC9 was associated with a 46% reduction in the odds of CHF (OR = 0.54, 95% CI: 0.50 – 0.59, P < 0.0001). In the fully adjusted Model 3, an increase of 10 points in LC9 was associated with a 24% reduction in the odds of CHF (OR = 0.76, 95% CI: 0.65 – 0.88, P < 0.001). When LC9 was included in the model as a four-category variable, after full adjustment (Model 3), compared with the quartile 1 group, the prevalence of CHF in the quartile 2 group (OR = 0.59, 95% CI: 0.44 – 0.80, P < 0.001), the quartile 3 group (OR = 0.56, 95% CI: 0.36 – 0.89, P = 0.01), and the quartile 4 group (OR = 0.35, 95% CI: 0.18 – 0.69, P = 0.003) all decreased significantly, and there was a trend of decreasing prevalence of CHF among the four groups (P for trend = 0.002).

**Table 4:** OR estimates for the association between LC9 and CHF.

		Model 0		Model 1		Model 2		Model 3	
		OR (95%CI)	*P-*value	OR (95%CI)	*P-*value	OR (95%CI)	*P-*value	OR (95%CI)	*P-*value
CHF~LC9	Per 10	0.54(0.50,0.59)	<0.0001	0.61(0.55,0.68)	<0.0001	0.61(0.54,0.70)	<0.0001	0.76(0.65, 0.88)	<0.001
	Quartile 1	reference		reference		reference		reference	
	Quartile 2	0.38(0.30,0.48)	<0.0001	0.43(0.33,0.55)	<0.0001	0.46(0.35,0.61)	<0.0001	0.59(0.44, 0.80)	<0.001
	Quartile 3	0.24(0.17,0.33)	<0.0001	0.34(0.24,0.48)	<0.0001	0.38(0.25,0.58)	<0.0001	0.56(0.36, 0.89)	0.01
	Quartile 4	0.08(0.05,0.14)	<0.0001	0.18(0.10,0.32)	<0.0001	0.22(0.12,0.41)	<0.0001	0.35(0.18, 0.69)	0.003
	*P* for trend		<0.0001		<0.0001		<0.0001		0.002

Notes: Model 0: Crude model. Model 1: Adjusted for age, sex, race, marital status, education, and poverty-income ratio. Model 2: Additionally adjusted for drinking and total energy intake. Model 3: Further adjusted for ASCVD, cancer, and hyperlipidemia.

Abbreviation: OR, odds ratio; CI, confidence interval; LC9, Life’s Crucial 9; CHF, congestive heart failure; eGDR, estimated glucose disposal rate; ASCVD, atherosclerotic cardiovascular disease.

As shown in **Figure 2C**, after fully adjusting for covariates, the RCS showed a negative linear relationship between LC9 and CHF (P for nonlinear = 0.116).

### Subgroup analysis

**Figure 3:**
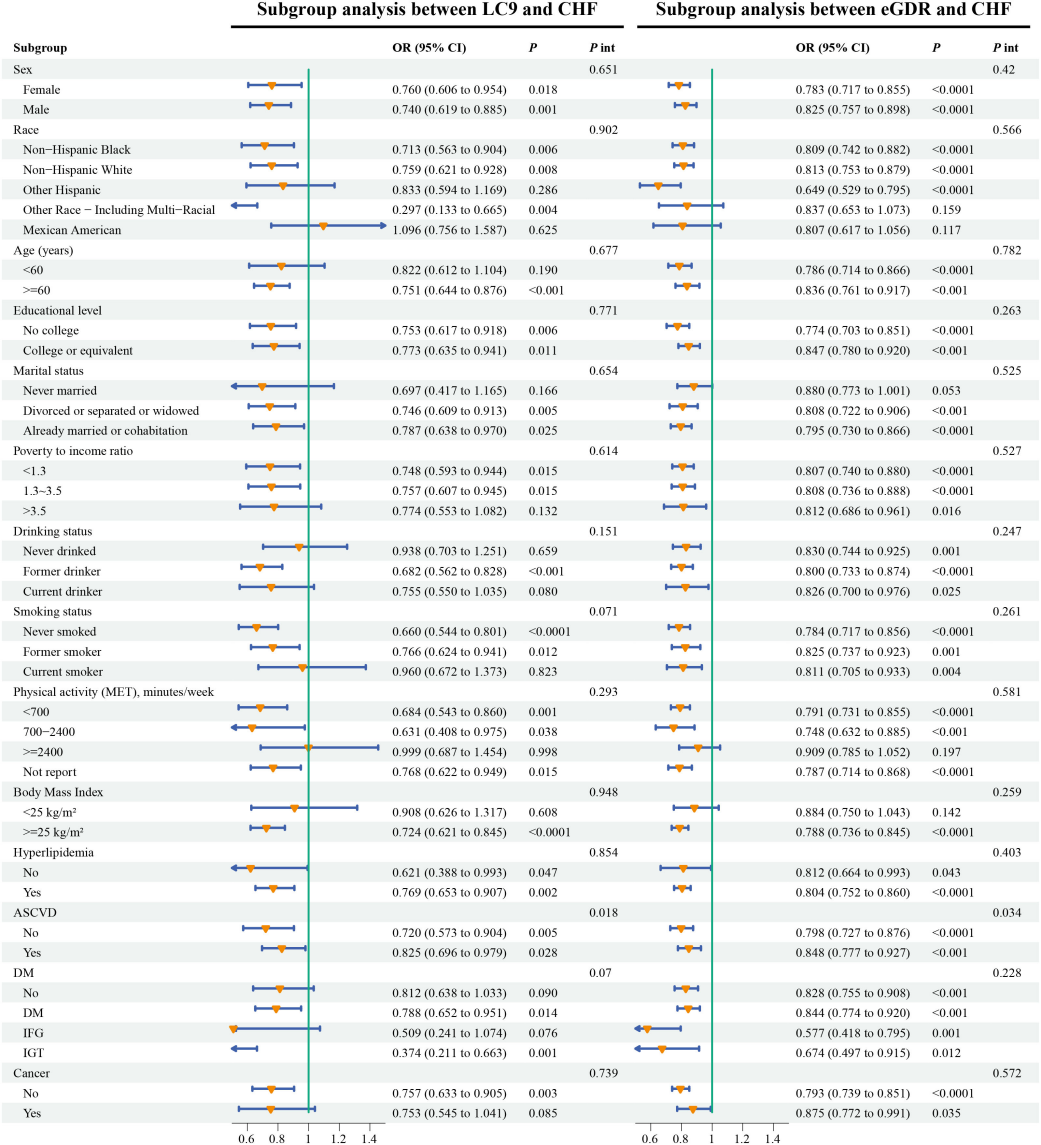
Weighted subgroup analyses of LC9 and eGDR with CHF. Models was adjusted for age, sex, race, marital status, education, poverty-to-income ratio, smoking, drinking, physical activity, dietary energy, BMI, cancer, DM, hyperlipidemia, and ASCVD. LC9, Life’s Crucial 9; eGDR, estimated glucose disposal rate; ASCVD, atherosclerotic cardiovascular disease; CHF, congestive heart failure; BMI, body mass index; DM, diabetes mellitus.

As shown in **Figure 3**, in the subgroup analysis, the associations between LC9 and CHF as well as between eGDR and CHF were weakened among patients with ASCVD, which might imply that ASCVD reduces the preventive effects of LC9 and eGDR on CHF.

### The mediating role of eGDR in the association between LC9 and CHF

**Figure 4:**
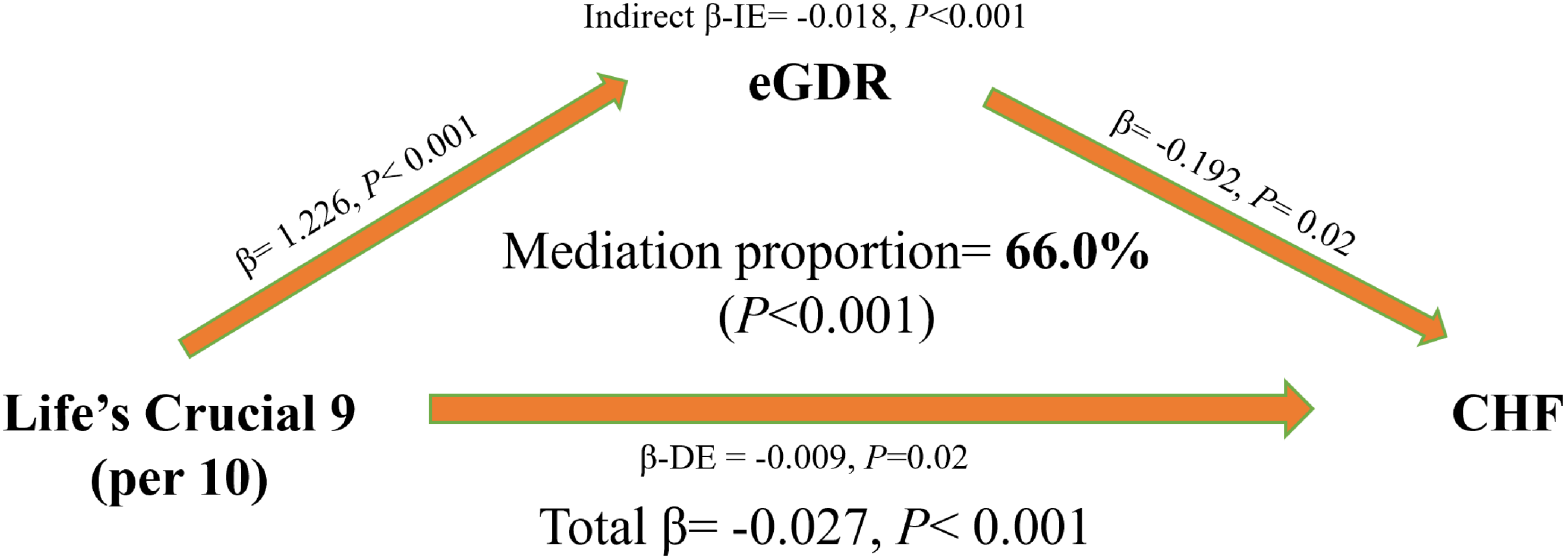
Mediation effect of eGDR in the association between LC9 and CHF. Bootstrap method with 5000 times repeated simulations was used to estimated the mediation effect of eGDR in the association between LC9 and CHF. Models was adjusted for age, sex, race, marital status, education, poverty-to-income ratio, drinking, total energy intake, ASCVD, cancer, and hyperlipidemia. LC9, Life’s Crucial 9; eGDR, estimated glucose disposal rate; ASCVD, atherosclerotic cardiovascular disease; CHF, congestive heart failure; BMI, body mass index; DM, diabetes mellitus.

**Figure 4** shows the results of the mediation analysis. After adjusting for confounding factors, eGDR was a significant mediator in the association between LC9 and CHF, with a mediation proportion of 66%.

## Discussion

In this nationally representative observational study involving 22,699 adult participants, we have demonstrated for the first time that LC9, the latest CVH indicator released by the AHA, is linearly and negatively associated with CHF. For every 10-point increase in LC9, the prevalence of CHF decreases by 24%. Moreover, LC9 is also independently and linearly positively associated with eGDR. For every 10-point increase in LC9, eGDR increases by 0.11. In addition, for every one-unit increase in eGDR, the odds of CHF decreases by 19%. Furthermore, in the subgroup analysis, the associations between LC9 and CHF as well as between eGDR and CHF were weakened among patients with ASCVD. In the mediation analysis, the results suggest that the association between LC9 and CHF is mainly mediated by eGDR, with a mediation proportion of 66%.

Several previous studies have reported the associations between CVH indicators in different periods and HF. Results from the Framingham Heart Study showed that for every one-unit increase in LS7, the incidence of HF would decrease by 23% (23). A cross-sectional study from NHANES revealed that the NCDs most closely related to LE8 were emphysema, CHF, and stroke (7). Yu et al. (24) analyzed the relationship between LE8 and NCDs among 170,000 samples in the UK Biobank and found that the proportion of patients with HF having poor CVH was as high as 34%, ranking among the top ten out of 44 NCDs. In 2023, a longitudinal cohort study from Finland demonstrated that the LS7 score was associated with a reduced risk of HF in the aging population, and participants with the best CVH scores had a 48% lower risk of HF (25). In 2024, prospective evidence from China also suggested that an ideal LE8 reduced the incidence of HF by 62% (26). The current study shows that the latest CVH indicator, LC9, is linearly and negatively associated with the prevalence of CHF. For every 10-point increase in LC9, the prevalence of CHF decreases by 24%. This study also indicates that among participants with ASCVD, the effect of LC9 on preventing HF is limited. This suggests that in diseases such as coronary heart disease and stroke, the progression of CHF seems to be more difficult to be halted. Participants with ASCVD often already have coronary atherosclerosis, resulting in long-term ischemia and hypoxia of the myocardium, and irreversible damage and necrosis of myocardial cells (27, 28). Even though the CVH represented by LC9 has certain positive effects, due to the relatively severe damage to myocardial cells, the ability of their functional recovery and remodeling is limited, thus restricting the preventive effect on HF. Secondly, myocardial ischemia and damage caused by ASCVD will initiate the process of ventricular remodeling, including myocardial cell hypertrophy, myocardial fibrosis, etc. (29, 30). These pathological changes will lead to thickening of the ventricular wall and enlargement of the cardiac chamber, which in turn affects the heart’s pumping function. Even if the cardiovascular protection mechanism related to LC9 plays a role, in the face of the already initiated ventricular remodeling process, its effect on stopping or reversing the remodeling is relatively weak, thereby limiting its preventive effect on HF.

Although IR is often regarded as a risk factor for CVD, research has shown that various CVDs can lead to disorders in the body’s glucose metabolism. Stress - induced hyperglycemia upon admission for acute coronary syndrome is associated with adverse outcomes, especially in non-DM patients (31). The occurrence of hypertension is a risk factor for type 2 diabetes mellitus (T2DM) and accelerates the development of IR (32). Additionally, myocardial ischemia can exacerbate insulin resistance through complex molecular pathways (33). Patients with coronary heart disease have also been found to exhibit characteristics of IR related to abnormal non - oxidative glucose or glycogen synthesis (34). These studies highlight the important role of CVH in glucose metabolism. Existing studies also suggest that good CVH can improve IR. One study that included 7,717 South Asians living in the United States found that poor CVH defined by LS7 increased homeostatic model assessment of insulin resistance (HOMA-IR) by approximately 1.5 times (17). In the Jackson Heart Study, for every one-item increase in LS7, the risk of DM decreased by 17% (35). Metabolic syndrome (MetS) is also known as insulin resistance syndrome. A study from NHANES showed an inverse dose-response relationship between the LE8 score and the prevalence of MetS (36). eGDR reflects the body’s ability to process glucose. The current study shows a strong positive relationship between LC9 and eGDR, indicating that maintaining good CVH is crucial for preventing IR.

The association between eGDR and CHF had not been explored before. However, previous studies have already confirmed the potential relationship between IR and HF. In the Framingham Heart Study, after adjusting for confounding factors, DM was associated with an increased risk of HF, especially among female participants (15). The emerging marker of IR, the TyG, has also been confirmed to be a risk factor for new-onset HF in the general population (16, 37). A meta-analysis also suggested that after considering traditional risk factors, a higher level of IR was associated with a higher risk of HF. This association existed in studies including both DM patients and non-DM patients (38). However, unlike other indicators, eGDR, as an alternative indicator of IR, is mainly used to assess T1DM. Up to now, the relationship between eGDR and CHF has been unclear; this study has, for the first time, confirmed in a large population that eGDR can reduce the prevalence of CHF. Mechanistically, HF is associated with left ventricular hypertrophy, manifested as an increase in wall thickness, an elevation in the left ventricular mass index, myocardial cell death, dilated cardiomyopathy, extracellular fibrosis, and functional abnormalities that affect diastolic and systolic functions. The development of cardiac dysfunction stems from both cardiovascular IR and peripheral and hepatic IR (39–41). The factors contributing to cardiac injury caused by IR include impaired calcium signaling, metabolic changes, mitochondrial dysfunction, oxidative stress, endoplasmic reticulum stress, and dysregulated myocardial - endothelial interactions (39). In the subgroup analysis, ASCVD weakened the protective effect of eGDR on CHF. Patients with ASCVD are often accompanied by multiple cardiovascular risk factors, such as hypertension, hyperlipidemia, and diabetes mellitus. These factors are intertwined and jointly promote damage to the cardiovascular system (42). These risk factors not only directly affect the structure and function of blood vessels and the myocardium but may also interfere with the insulin signaling pathway through complex mechanisms and aggravate insulin resistance. Therefore, in patients with ASCVD, the relationship between insulin resistance represented by eGDR and CHF is masked or confounded by many other factors, weakening the direct association between them.

The biological basis for the association between ideal CVH and a lower risk of HF is still unclear. Previously, the study by Li et al. (23) explored the intermediate mechanism between CVH represented by LS7 and HF. Through mediation analysis, they found that seven metabolites mediated the relationship between LS7 and HF. These metabolites were mainly involved in three main pathways, including alanine, glutamine and glutamate metabolism, citric acid cycle metabolism, and glycerolipid metabolism (23). This study found that the association between CVH represented by LC9 and CHF was mainly mediated by eGDR, with a mediation proportion of 66%, which was highly consistent with the study by Li et al. (23). Alanine, glutamine and glutamate metabolism, citric acid cycle metabolism, and glycerolipid metabolism are all closely related to insulin resistance. The metabolism of amino acids such as alanine, glutamine and glutamate in the body is interrelated with glucose metabolism. For example, alanine plays an important role in gluconeogenesis. It can generate pyruvate through transamination and then participate in the gluconeogenesis pathway, affecting blood glucose levels (43, 44). When the body is in a state of insulin resistance, glucose metabolism is disrupted, which may lead to abnormal metabolism of amino acids such as alanine. Conversely, abnormal alanine metabolism may also feedback and affect the action of insulin, aggravating insulin resistance (45, 46). The citric acid cycle is an important metabolic pathway for energy production in cells, and the adenosine triphosphate (ATP) it produces is the direct energy source for cell activities (47). In the state of insulin resistance, the uptake and utilization of glucose by cells are impaired, leading to an imbalance in energy metabolism. The operating efficiency of the citric acid cycle may be affected, thereby reducing the production of ATP (48–50). The decrease in intracellular ATP levels will feedback and affect the insulin - signaling pathway, reducing insulin sensitivity and forming a vicious cycle (50). Abnormal glycerolipid metabolism is one of the important characteristics of insulin resistance. When glycerolipid metabolism is disrupted, the lipid breakdown and fatty acid release in adipocytes increase, and excessive fatty acids will spill over to other tissues, such as the liver and muscles, leading to ectopic lipid deposition (51). In addition, from the perspective of metabolic pathways, LC9 encompasses multiple CVH elements, such as the comprehensive management and control status of blood pressure, blood lipids, and blood glucose. When the CVH reflected by LC9 is at an unsatisfactory level, the body’s glucose metabolism is often the first to be affected, resulting in an increase in IR and subsequently leading to an imbalance in blood glucose homeostasis (52). At this time, eGDR, as an indicator measuring the body’s ability to uptake, utilize, and store glucose, will undergo significant changes accordingly. A decrease in its value indicates a lower glucose processing efficiency. Moreover, a long-term environment of high blood glucose and inefficient glucose metabolism will prompt the accumulation of a series of adverse metabolic products in the body, such as advanced glycation end products (AGEs). These products can directly modify cardiomyocyte proteins, affect the contraction and relaxation functions of cardiomyocytes, and activate inflammatory signaling pathways to induce a chronic inflammatory response in the myocardial tissue, gradually impairing the myocardial structure and function and ultimately increasing the risk of CHF (53, 54). From the perspective of neuroendocrine regulation, an unfavorable LC9 status may trigger the body’s stress response, leading to over - activation of the sympathetic nervous system. On one hand, this causes an increase in blood pressure, exacerbating the cardiac after - load. On the other hand, a large amount of catecholamines are secreted, which interfere with insulin signal transduction, further deteriorating glucose metabolism and causing eGDR to continuously decline (55). Meanwhile, sustained sympathetic excitation also activates the renin - angiotensin - aldosterone system (RAAS), resulting in water and sodium retention and vasoconstriction. This increases both the cardiac pre - load and after - load. Under long - term high - pressure loading, cardiomyocytes gradually hypertrophy and undergo fibrosis. As a result, cardiac function deteriorates continuously, ultimately leading to CHF (56, 57). During this process, the decrease in eGDR is always accompanied by glucose metabolism disorders, and it acts in synergy with the deterioration of CVH associated with LC9, accelerating the progression towards CHF. Considering the association between oxidative stress and inflammatory response, the cardiovascular risk factors involved in LC9, such as dyslipidemia and hypertension, can trigger an increase in the level of oxidative stress in the body and generate a large number of reactive oxygen species (ROS). ROS can not only directly damage organelles such as mitochondria in cardiomyocytes, affecting myocardial energy metabolism, but also activate the release of various inflammatory cytokines, such as tumor necrosis factor-α (TNF-α) and interleukin-6 (IL-6), creating a pro - inflammatory microenvironment (58, 59). In this environment, intracellular signal transduction pathways are disrupted, further affecting the action of insulin and causing eGDR to decline. Moreover, the continuous progression of the inflammatory response will accelerate cardiomyocyte apoptosis and myocardial tissue remodeling, ultimately leading to heart failure, that is, the occurrence of CHF. In this series of complex pathophysiological processes, eGDR is at the key node of the interaction network of metabolism, neuroendocrine, and oxidative stress and inflammatory response, closely linking LC9 and CHF together (60).

Mental health plays a pivotal role in overall health assessment. Although LC9 was initially constructed based on the LE8, its connotation has been further enriched and expanded after incorporating the dimension of mental health. In contemporary society, mental states such as psychological stress, anxiety, and depression are closely linked to numerous health issues like CVD. A large number of studies have shown that being in a negative mental state for a long time can affect the physiological functions of the body through multiple pathways such as the neuroendocrine and immune systems, and subsequently disrupt the normal operation of the cardiovascular system (61–63). For example, the dysregulation of cortisol secretion caused by chronic stress may lead to elevated blood pressure and abnormal blood lipids, indirectly increasing the risk of CVD (64, 65). Therefore, incorporating mental health into the LC9 scoring system is of great practical significance for accurately assessing an individual’s cardiovascular health risk. The weight of mental health in the LC9 scoring system is a complex issue worthy of in-depth discussion. On the one hand, from a theoretical perspective, since the influence pathways of mental health on physical health are multiple and indirect, it is difficult to simply quantify its exact contribution. Different mental states produce varying intensities and durations of impact. For instance, an acute anxiety attack may cause a rapid heart rate and blood pressure fluctuations in the short term, while long-term depressive mood erodes overall health more insidiously and profoundly (66, 67). On the other hand, from the perspective of actual data collection, compared with traditional physiological indicators, it is more difficult to measure mental health indicators, with many problems such as subjective cognitive differences and limitations of measurement tools.

In conclusion, by leveraging the population-based research in the NHANES database, we comprehensively explored the association among LC9, eGDR, and CHF. These findings have provided valuable insights into the underlying relationships and mechanisms of these factors. From a clinical perspective, clinicians could consider incorporating LC9 assessment into routine examinations of CVH, especially for individuals with risk factors such as diabetes or a family history of heart disease. Monitoring the changes of eGDR over time can also serve as an early warning sign for the emergence of cardiovascular health problems. In addition, lifestyle modifications can be tailored according to an individual’s LC9 and eGDR status, such as promoting healthy diets and regular physical activities, to optimize CVH. Public health initiatives can be designed to raise public awareness of these factors and encourage preventive actions among the general population, ultimately aiming to reduce the burden of CVD. Future research could focus on several key directions. Firstly, it is necessary to conduct longitudinal studies to further validate and track the changes in these associations over time. This will help to establish more conclusive causal relationships and understand the dynamic nature of how LC9 and eGDR interact with CHF risk factors as individuals age. Secondly, it would be beneficial to design studies specifically for the Chinese population, incorporating regionally specific dietary patterns, lifestyle habits, and genetic predispositions. By doing so, we can deepen our understanding of these relationships and develop more targeted prevention and intervention strategies. Thirdly, investigating potential mediating and moderating factors that have not been explored in this study may reveal other pathways through which LC9 and eGDR affect the prevalence of CHF. For example, exploring the role of environmental pollutants or psychological stress in modulating these associations might open up new avenues for research.

## Advantages and limitations

In this large-scale cross-sectional study, we have explored for the first time the linear negative association between LC9 and CHF, and eGDR is a significant mediator factor. This study has a large sample size and comprehensively considers multiple confounding factors, so the results are relatively reliable.

However, several limitations need to be disclosed. Firstly, this study uses cross-sectional survey data, which cannot demonstrate causal relationships. Due to the lack of dynamic information in the time dimension, we are unable to definitively determine the causal direction. It is difficult to distinguish whether IR initiates changes in cardiovascular function first, subsequently leading to an increased risk of disease, or whether the potential hidden dangers of CVD prompt the body’s metabolic regulation to become unbalanced and induce IR. Especially when it comes to complex physiological and pathological processes, such as the interactive feedback between the insulin signaling pathway and the cardiovascular system. Cross-sectional data makes it hard to accurately analyze the sequence of events, restricting the in-depth interpretation of the causal mechanism. In terms of future research directions, it is necessary to rely on longitudinal tracking data or interventional experiments to further clarify the causal links among LC9, IR, and CHF, filling the gaps left by existing cross-sectional studies. Secondly, although many covariates have been adjusted, potential confounding factors may still not have been taken into account. For example, occupational types and the resulting workplace exposure factors may interfere with the relationships among LC9, eGDR, and CHF through multiple potential pathways. Different occupations vary in terms of the intensity of physical labor, psychological stress load, and exposure to chemical substances or physical radiation. These are all highly likely to be crucial variables affecting CVH and the occurrence and development of diseases, yet they have not been fully considered in this study. In the future, if relevant research is carried out, efforts should be made to improve the collection of occupational information. For example, occupational categories should be classified in detail, and the degree of exposure to harmful substances in the workplace should be accurately quantified, so as to more precisely analyze the impact mechanism of the combined effects of various factors on the risk of CVD and fill this gap in current research. Thirdly, as Ge et al. (9) acknowledged, a more comprehensive assessment of the psychological factor indicators of LC9 is needed in the future, such as taking into account psychological disorders like anxiety. Fourthly, in this study, some crucial information did rely on the self-reports of the participants, such as aspects related to lifestyle habits (smoking, drinking, exercise frequency, etc.), dietary intake, and details of medical history. This method of data collection poses the risk of introducing reporting bias. On the one hand, participants may, due to memory lapses, be unable to accurately recall the details of their behaviors or events over a relatively long period in the past. For example, it is difficult for them to precisely remember the number of weekly exercise sessions or the specific amount of alcohol consumed each time in the past year. On the other hand, due to psychological factors like social desirability, some participants may, either intentionally or unintentionally, modify certain sensitive information. For instance, they may underreport the amount of smoking or exaggerate the proportion of healthy diet, thus causing the collected data to deviate from the real situation. In future research, we will explore how to incorporate more objective measurement methods, such as using wearable devices to monitor exercise and sleep and detecting biomarkers to evaluate the effect of dietary intake, in order to make up for the deficiencies of relying solely on self-reports, improve data quality, and subsequently enhance the reliability of the research. Finally, the current research focuses on the adult population in the United States. Although this sample has provided information on the associations among LC9, eGDR, and CHF, it is undeniable that expanding the research scope to groups with different demographic characteristics, including different races, ethnicities, age groups, and geographical regions, is expected to uncover more in-depth information. For example, significant differences exist in genetic backgrounds, lifestyles, and dietary habits among different races, and these factors are highly likely to affect the CVH elements encompassed by LC9 as well as the metabolic level reflected by eGDR. Compared with Caucasian populations, African American populations may, due to a higher proportion of traditional high-calorie diet intake and a relatively lack of regular exercise habits, exhibit unique distribution characteristics in each dimension of LC9 and eGDR values, and thus establish an association pattern with CVD risk that is different from other races (68–70). From the age dimension, the physical functions of children, adolescents, middle-aged people, and the elderly are at different stages, and the development and metabolic characteristics of the cardiovascular system also vary (71, 72). The high metabolic level of young people may have a positive moderating effect on eGDR, so that under the same lifestyle intervention, the change trend of their eGDR is completely different from that of the elderly, and this will in turn have a chain reaction on the interaction relationship with LC9, affecting the overall assessment of CVH. Exploring the differences under different healthcare systems is equally crucial. In developed countries like the United States, medical resources are relatively abundant, and the preventive healthcare system is relatively complete. The public has a strong awareness of early screening and intervention for cardiovascular diseases, which may enable the LC9 and eGDR indicators of the population to be effectively monitored and regulated in the early stage of the disease process. In contrast, in some developing countries, medical resources are scarce and the awareness of healthcare is weak. CVD is often not discovered until it has reached a relatively serious stage. At this time, the states of LC9 and eGDR and the relationship between them may have undergone qualitative changes. By comparing these differences under different healthcare systems, more targeted bases can be provided for the formulation of global CHF prevention and control strategies.

## Conclusion

In conclusion, this study shows that both LC9 and eGDR are negatively associated with the prevalence of CHF, and eGDR significantly mediates the association between LC9 and CHF. It will be necessary to verify our findings in longitudinal cohorts in the future.

## Data Availability

Publicly available datasets were analyzed in this study. This data can be found here: https://wwwn.cdc.gov/nchs/nhanes/.
